# Prevalence of self-reported abdominal symptoms among 50–74-years-old men and women eligible for colorectal cancer screening –a cross-sectional study

**DOI:** 10.1186/s12885-021-08657-z

**Published:** 2021-08-10

**Authors:** Mette Bach Larsen, Heidi Heinsen Bachmann, Bo Søborg, Tinne Laurberg, Katrine J. Emmertsen, Søren Laurberg, Berit Andersen

**Affiliations:** 1 University Research Clinic for Cancer Screening, Department of Public Health Programmes, Randers Regional Hospital, Skovlyvej 15, NO DK-8930 Randers, Denmark; 2 Steno Diabetes Center Aarhus, Aarhus University Hospital, Hedeager 3, DK-8200 Aarhus N, Denmark; 3 Department of Surgery, Aarhus University Hospital, Palle Juul-Jensens Boulevard 35, DK-8200 Aarhus N, Denmark; 4 Department of Surgery, Randers Regional Hospital, Skovlyvej 1, NO DK-8930 Randers, Denmark; 5 Department of Clinical Medicine, Aarhus University, Incuba Skejby, Building 2, Palle Juul-Jensens Boulevard 82, DK-8200 Aarhus N, Denmark

**Keywords:** Abdominal symptoms, Prevalence, Low anterior resection syndrome score (LARS), Patient assessment of constipation-symptoms (PAC-SYM), Mass screening

## Abstract

**Background:**

Screening is defined as the identification of unrecognized disease in an apparently healthy population. Symptomatic individuals are recommended to contact a physician instead of participating in screening. However, in colorectal cancer (CRC) screening this approach may be problematic as abdominal symptoms are nonspecific. This study aimed at identifying the prevalence of self-reported abdominal symptoms among screening-eligible men and women aged 50–74 years.

**Methods:**

This cross-sectional survey study included 11,537 individuals aged 50–74 years invited for CRC screening from 9 to 23 September 2019. Descriptive statistics of responders experiencing alarm symptoms of CRC, Low Anterior Resection Syndrome Score (LARS) and the Patient Assessment of Constipation-Symptoms (PAC-SYM) were derived. The association between abdominal symptoms and demographic and socioeconomic variables were estimated by prevalence ratio (PR) using a Poisson regression model with robust variance.

**Results:**

A total of 5488 respondents were included. The respondents were more likely women, of older age, Danish, cohabiting and had higher education and income level compared to non-respondents.

Abdominal pain more than once a week was experienced by 12.0% of the respondents. Of these, 70.8% had been experiencing this symptom for >1 month. Fresh blood in the stool was experienced by 0.7% and of these 82.1% for >1 month. About one third of those experiencing alarm symptoms more than once a week for >1 month had not consulted a doctor. A total of 64.1% of the respondents had no LARS, 21.7% had minor LARS and 14.2% had major LARS. The median PAC-SYM score was 0.33 (Interquartile range (IQR): 0.17;0.75), the median abdominal score was 0.50 (IQR: 0.00;1.00), median rectal score 0.00 (IQR:0.00;0.33) and median stool score 0.40 (IQR: 0.00;0.80). Men and those aged 65–74 reported less symptoms than women and those aged 50–64 years, respectively.

**Conclusions:**

This study illustrated that abdominal symptoms were frequent among screening-eligible men and women. This should be taken into account when implementing and improving CRC screening strategies. A concerning high number of the respondents experiencing alarm symptoms had not consulted a doctor. This calls for attention to abdominal symptoms in general and how those with abdominal symptoms should participate in CRC screening.

**Supplementary Information:**

The online version contains supplementary material available at 10.1186/s12885-021-08657-z.

## Background

Accounting for approximately 10% of all incident cancers and 10% of all cancer-related deaths, colorectal cancer (CRC) constitutes a significant part of the cancer burden among both men and women [1]. To reduce morbidity and mortality from CRC, screening programs for asymptomatic populations at average risk of developing CRC have been implemented in many countries [2]. Screening is defined as the presumptive identification of unrecognized disease in an apparently healthy and asymptomatic population according to the World Health Organization. Information material in CRC screening therefore often recommends symptomatic individuals to contact a physician instead of participating in screening. However, this approach may not be optimal for several reasons. First, symptom interpretation is a multidimensional construct influenced by social and cultural settings as well as psychological processes [3]. The same bodily sensations may be interpreted differently from one individual to another depending on e.g. sex, age and context. Second, abdominal symptoms are frequent in the general population [4, 5]. In Europe, as many as 10% of consultations in general health care are due to abdominal symptoms but only 0.3% of these result in patients being diagnosed with an incident abdominal cancer within 6 months [6]. Third, it is well-known that alarm symptoms of CRC are unspecific and yield low positive predictive values of cancer [7, 8]. Finally, as many as 25% of CRCs diagnosed within 1 year after invitation are diagnosed outside the screening program, primarily by referral from the general practitioner [9]. Further, many organized CRC screening programs suffer from suboptimal participation rates [10].

Knowledge about self-reported symptoms among screening eligible men and women is an important starting point for understanding CRC screening behavior and optimize the screening programs. Thus, this study aimed at identifying the frequency of self-reported abdominal symptoms among screening-eligible men and women aged 50–74 years assessed as frequency of alarm symptoms, proportion experiencing symptoms for 1 month or longer and proportion who has consulted a doctor. Further, frequency of symptoms related to Lower Anterior Resection Syndrome (LARS) and constipation was reported.

## Methods

### Setting

This study took place in the Central Denmark Region which is inhabited by 1.3 million people including both urban and rural areas and the second largest city in Denmark (Aarhus) [11].

The Danish CRC screening program is initiated nationally and administered regionally. All residents aged 50–74 years are invited biennially. Invitations are sent to their home address with a screening kit (OC Sensor system (Eiken Chemical Company, Tokyo, Japan)) and a pre-addressed, pre-paid return envelope. If a fecal sample is not returned within 45 days, one reminder is sent. Participation and any subsequent treatment are free of charge for all citizens [12].

Digital communication with the health care system is mandatory in Denmark for citizens aged 15 years and older through a secure email platform [13]. In 2019, 5.9% of the 55–64 year olds and 12.3% of the 65–74 year olds were exempt from digital communication and received all communication by postal mail [14].

### Study design and population

**Fig. 1:**
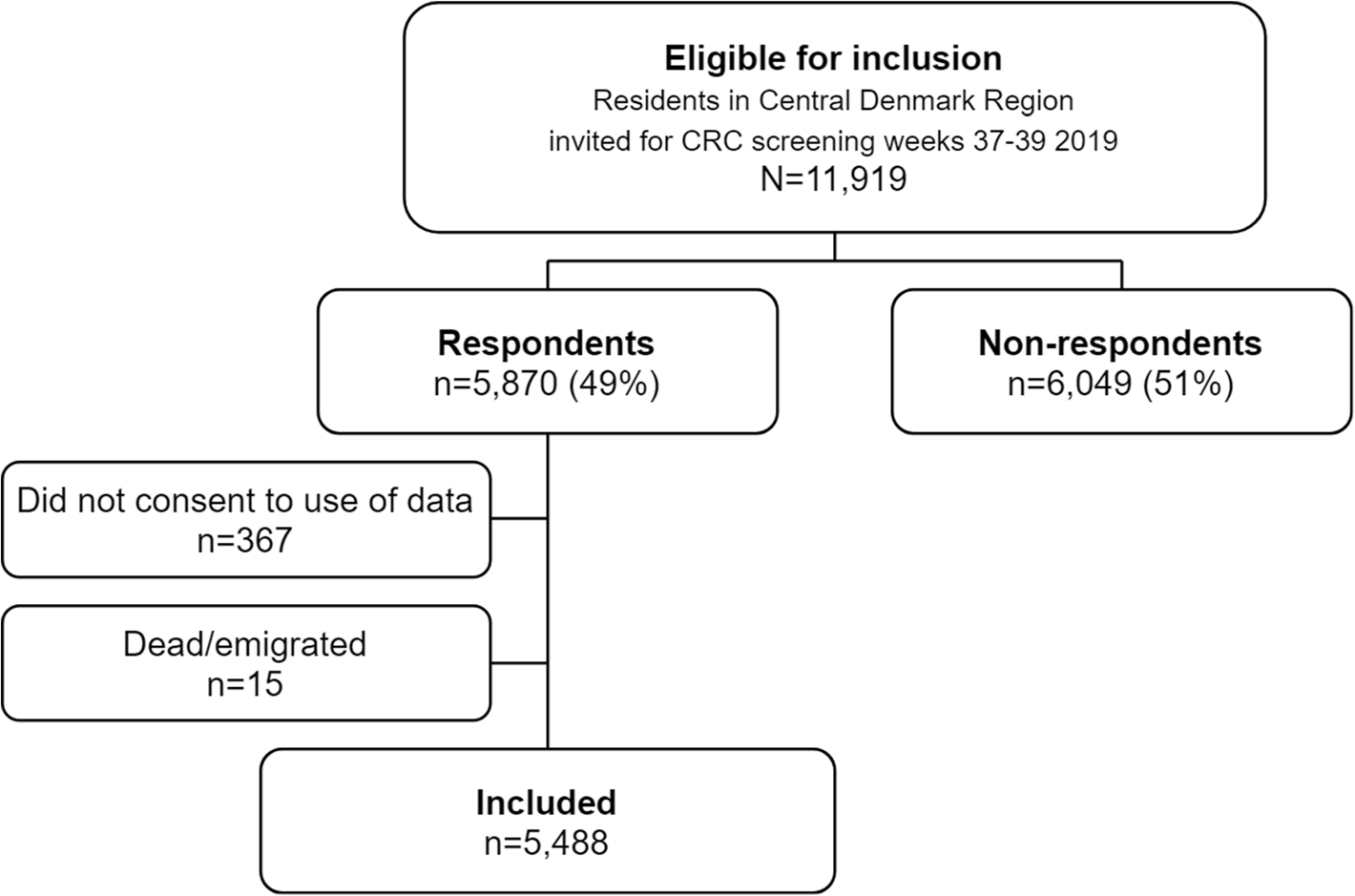
Flowchart illustrating the study inclusion

The study was conducted as a cross-sectional study using survey and register data. The study population consisted of 11,537 women and men in the age group 50–74 years living in Central Denmark Region and about to be invited for CRC screening in the period 9–23 September 2019 which was three standard weeks in the screening program. The study population was identified in the regional Invitation and Administration Module of the screening program. We excluded individuals participating in the survey but not consenting to use of register data, and we excluded those who died or emigrated from the Central Denmark Region before register data were collected (Fig. 1).

### Data

The research group developed a questionnaire with ad hoc questions to explore the experience of six symptoms: abdominal pain, mucus, fresh or old blood in stool, unexplained weight loss and unexplained tiredness. In May 2019, these six questions were quantitatively pilot tested among 100 randomly selected individuals of which 39 returned the questionnaire. Of those, 20 individuals agreed to a qualitative pilot test by participating in semi-structured telephone interviews. The interviews explored whether the participants understood the questions and the answering categories, experienced abdominal symptoms not included in the questionnaire, and their general acceptance of the questionnaire. Neither the quantitative nor the qualitative pilot test gave rise to changes in the six questions.

Abdominal pain, mucus in stool, fresh blood in stool and dark/black stool occurring at least once a week were defined as presence of symptoms. If a symptom was present, respondents were asked to state how long it had been present. Unexplained weight loss within the last months and unexplained tiredness within the last 4 weeks were defined as present if it was experienced some or a lot.

The questionnaire also included two validated scales to measure symptoms from the bowel: The Low Anterior Resection Syndrome (LARS) score and the Patient Assessment of Constipation-Symptoms (PAC-SYM). The LARS score was originally developed to describe the constellation of symptoms after receiving sphincter-preserving surgery for rectal cancer [15]. The scale consists of five items. The first two items address frequency of incontinence for flatus and liquid stool, the third item describes frequency of stool and the last two items describes fragmentation of stool and urgency. The original LARS scoring system had three answering categories for each item. Because these data were to be used in a clinical setting besides this study, three answering categories were not sufficiently detailed. The three original answering categories corresponded to the following answering categories in our questionnaire: Never = ‘Never’; Less than once per week = ‘Less than once a month’, ‘Once a month’ and ‘More than once a month but not every week’; More than once per week = ‘At least once a week but not every day’ and ‘At least once a day’. According to the scoring manual, each option has a weight value. By adding these, a total score value was created in the range of 0 to 42 corresponding to three categories based on severity of symptoms: no LARS (0 to 20), minor LARS (21 to 29) and major LARS (30 to 42).

The PAC-SYM is a self-reporting instrument developed to describe the patients’ experience of constipation symptoms within 2 weeks and compare them over time [16]. The scale contains 12 items divided into three subcategories: stool symptoms, rectal symptoms and abdominal symptoms. Each item is scored from 0 (absence of symptoms) to 4 (worst symptom severity). Symptoms were defined as present if respondents answered serious or very serious (3 or 4). The total score was divided with the number of items, creating a total mean score from 0 to 4.

Table 1 presents questions and response categories for all questions.

**Table 1:** Demographic and socioeconomic characteristics of responders and non-responders (*N* = 11,537)

	Responders n (%)	Non-responders n (%)
**All**	5488 (47.6)	6049 (52.4)
**Sex**		
Men	2702 (49.2)	3176 (52.5)
Women	2786 (50.8)	2873 (47.5)
**Age** (mean years (SD))	62.6 (7.33)	61.7 (7.69)
Men		
50-64y	1398 (51.7)	1986 (62.5)
65-74y	1304 (48.3)	1190 (37.5)
Women		
50-64y	1547 (55.5)	1635 (56.9)
65-74y	1239 (44.5)	1238 (43.1)
**Origin**		
Danish	5280 (96.2)	5380 (88.9)
Western	121 (2.20)	197 (3.26)
Non-Western	69 (1.26)	398 (6.58)
Missing	18 (0.33)	74 (1.22)
**Marital status**		
Cohabiting	4273 (77.9)	3872 (64.0)
Living alone	1197 (21.8)	2103 (34.8)
Missing	18 (0.33)	74 (1.22)
**Education**		
≤ 10y	1054 (19.2)	1671 (27.6)
11-15y	2791 (50.9)	2935 (48.5)
> 15y	1582 (28.8)	1245 (20.6)
Missing	61 (1.11)	198 (3.27)
**Income**		
Low tertile	1322 (24.1)	2492 (41.2)
Middle tertile	2020 (36.8)	1797 (29.7)
High tertile	2128 (38.8)	1686 (27.9)
Missing	18 (0.33)	74 (1.22)

Differences between the groups were tested using Pearson’s Chi-squared test. *P* < 0.001 for all variables except age groups for women (*P* = 0.295)

Data on socio-demographic position were obtained from Statistics Denmark [17]. Using Statistics Denmark’s classification, origin was categorized by country of origin as either Danish, Western (EU, Andorra, Australia, Canada, Iceland, Liechtenstein, Monaco, New Zealand, Norway, San Marino, Switzerland, and the USA), or non-Western (others). Marital status was classified as cohabitating or living alone. Educational attainment was classified according to UNESCO’s classification as low (≤10 years), middle (11–15 years), or higher education (> 15 years) [18]. Family disposable household income based on the OECD-modified equivalence scale was used as an income measure [19]. Using tertiles, income was categorized as low (lowest 33%), middle (33–66%) or high (highest 33%).

Age and sex was identified from the unique personal identification number given to all residents in Denmark at birth or upon immigration [20]. This number was also used to combine survey and register data at an individual level.

### Data collection

The questionnaire was set up in REDCap™ (Research Electronic Data Capture) hosted at Aarhus University. REDCap is a secure web-based software platform designed to support data capture for research studies [21, 22]. The questionnaires were sent electronically using the secure platform on three consecutive Mondays from 9 to 23 September 2019. Those exempt from digital communication received the questionnaire by postal mail but had to type the link to the questionnaire in order to respond. Non-responders received a reminder after 2 weeks. Data collection was closed 1 November 2019.

### Statistical analyzes

Descriptive statistics were used to compare demographic and socioeconomic characteristics of responders and non-responders to the questionnaire. Differences between the two groups were tested using Pearson’s Chi-Squared test.

The proportion of responders experiencing alarm symptoms of CRC was reported as well as the proportion experiencing symptoms for >1 month. Out of those, the proportion who has seen a doctor for their abdominal symptoms was reported. As a sensitivity analysis, the definition of when alarm symptoms were present was changed to those experiencing symptoms more than once a month instead of at least once a week.

Proportions of responders experiencing no, minor and major LARS was reported along with the proportion experiencing each symptom more than once a week.

The median PAC-SYM score along with the median sub-scores for abdominal, rectal and stool symptoms were reported. Finally, the proportions of respondents experiencing each of the symptoms were reported.

The association between abdominal symptoms and demographic and socioeconomic variables were estimated by prevalence ratio (PR) using Poisson regression model with robust variance [23].

When relevant, 95% confidence interval (CI) or interquartile range (IQR) was calculated when relevant. Further, the results were presented for the total study population and stratified by sex and age groups (50–64 years and 65–74 years). Non-responders in each of the applied scales were excluded.

All analyzes were conducted using STATA version 16 (STATA Corp., College Station, Tex., USA).

## Results

A total of 11,919 men and women were eligible for inclusion and 5870 answered the questionnaire corresponding to a response rate of 49%. Subsequently, 367 respondents were excluded because they did not consent to use of register data and 15 respondents died or emigrated before collection of register data. Thus, 5488 (46%) were included in the analyzes (Fig. 1). Compared to non-respondents, the respondents were more likely women, of older age, Danish, cohabiting, and had higher education and income level (Table 1).

### Alarm symptoms

Overall, abdominal pain at least once a week and unexplained tiredness within the last 4 weeks were the most commonly experienced symptoms, reported by 13.1% (95% CI: 12.2;14.0) and 12.0% (95% CI: 11.1;12.8), respectively. Fresh blood in the stool was experienced by 0.7% (95% CI: 0.5;1.0) and of these 82.1% (95% CI: 66.5;92.5) had been experiencing this for >1 month. The combination of at least one of the other symptoms with unexplained weight loss or tiredness was experienced by 0.6% (95% CI: 0.4;0.9) and 5.0% (95% CI: 4.4;5.6), respectively (Table 2).

**Table 2:** Proportion of respondents experiencing alarm symptoms of colorectal cancer and proportion having experienced symptoms for one month or longer (95% confidence interval (CI)) among men and women aged 50–74 years (*N* = 5488)

	Total % (95 CI)	Women % (95 CI)			Men % (95 CI)		
	*N =* 5488	50-64y *n =* 1557	65-74y *n* = 1239	All *n* = 2786	50-64y *n* = 1398	65-74y *n* = 1304	All *n* = 2702
Abdominal pain^a^	12.0 (11.1;12.8)	17.2 (15.3;19.2)	13.4 (11.5;15.4)	15.5 (14.2;16.9)	9.5 (8.0)	7.0 (5.7;8.5)	8.3 (7.3;9.4)
One month or longer	78.5 (75.1;81.6)	80.5 (75.2;85.0)	75.8 (68.5;82.1)	78.7 (74.5;82.4)	76.7 (68.6;83.6)	80.2 (70.6;87.8)	78.1 (72.1;83.4)
Seen doctor	63.2 (58.9;67.4)	N/A	N/A	63.4 (58.0;68.6)	N/A	N/A	62.9 (55.2;70.0)
Mucus in stool^a^	1.7 (1.4;2.1)	2.3 (1.6;3.2)	1.5 (0.9;2.4)	2.0 (1.5;2.6)	1.3 (0.8;2.0)	1.8 (1.1;2.6)	1.5 (1.1;2.1)
One month or longer	79.2 (69.7;86.8)	77.8 (60.8;89.9)	63.2 (38.4;83.7)	72.7 (59.0;83.9)	94.4 (72.7;99.9)	82.6 (61.2;95.0)	87.8 (73.8;95.9)
Seen doctor	67.1 (55.4;77.5)	N/A	N/A	65.0 (48.3;79.4)	N/A	N/A	69.4 (51.9;83.7)
Fresh blood in stool^a^	0.7 (0.5;1.0)	1.1 (0.6:1.8)	0.4 (0.1;0.9)	0.8 (0.5;1.2)	0.7 (0.3)	0.5 (1.3)	0.6 (0.4;1.0)
One month or longer	82.1 (66.5;92.5)	82.4 (56.6;96.2)	100 (N/A)	86.4 (65.1;97.1)	80.0 (44.4;97.5)	71.4 (29.0;96.3)	76.5 (50.1;93.2)
Seen doctor	65.6 (46.8;81.4)	N/A	N/A	63.2 (38.4;83.7)	N/A	N/A	69.2 (38.6;90.9)
Very dark/black stool^a^	2.0 (1.6;2.4)	2.1 (1.4;2.9)	2.1 (1.4;3.1)	2.1 (1.6;2.7)	1.8 (1.2;2.6)	2.0 (1.3;2.9)	1.9 (1.4;2.5)
One month or longer	67.0 (57.3;75.7)	56.3 (37.7;73.6)	69.2 (48.2;85.7)	62.1 (48.4;74.5)	68.0 (46.5;85.1)	76.9 (56.4;91.0)	72.5 (58.3;84.1)
Seen doctor	43.8 (32.2;55.9)	N/A	N/A	41.7 (25.5;59.2)	N/A	N/A	45.9 (29.5;63.1)
Unexplained weight loss^b^	1.6 (1.3;1.9)	1.1 (0.6;1.8)	1.5 (0.9;2.3)	1.3 (0.9;1.7)	2.0 (1.3)	1.8 (1.1;2.6)	1.9 (1.4;2.5)
Unexplained tiredness^c^	13.1 (12.2;14.0)	15.6 (13.9;17.6)	10.0 (8.4;11.8)	13.1 (11.9;14.4)	15.4 (13.5;17.4)	10.4 (8.8;12.1)	13.0 (11.7;14.3)
At least one abdominal symptom *and* weight loss	0.6 (0.4;0.9)	0.6 (0.3;1.9)	0.7 (0.3;1.4)	0.7 (0.4;1.1)	0.6 (0.3;1.2)	0.5 (0.2;1.0)	0.6 (0.3;0.9)
At least one abdominal symptom *and* tiredness	5.0 (4.4;5.6)	6.5 (5.3;7.8)	5.0 (3.9;6.4)	5.8 (5.0;6.7)	4.7 (3.7;6.0)	3.4 (2.5;4.5)	4.1 (3.4;4.9)

^a^Proportion experiencing this symptom at least once a week

^b^Proportion reporting some or a lot unexplained weight loss during the last months

^c^Proportion reporting some or a lot unexplained tiredness within the last four weeks

Changing the definition of symptom experience to include those who had experienced the symptoms more than once a month instead of at least once a week, increased the proportion reporting the symptoms but not the described patterns in symptom experience (data not shown).

### Low anterior resection syndrome score (LARS)

A total of 64.1% (95% CI: 62.8;65.3) of the respondents had no LARS, 21.7% (95% CI: 20.6;22.8) had minor LARS and 14.2% (95% CI: 13.3;15.2) had major LARS. Incontinence for flatus or feces was reported by 25.0 and 3.2%, respectively. Abnormal frequency of bowel movements, fragmentation of stools and urgency were reported by 24.1% (95% CI: 23.0;25.2), 12.1% (95% CI: 11.3;13.0) and 13.7% (95% CI: 12.8;14.7), respectively (Table 3).

**Table 3:** Proportion of respondents experiencing no, minor and major Low Anterior Resection Syndrome (LARS) and proportion experiencing symptoms of incontinence for flatus and faeces; abnormal frequency of bowel movements; fragmentation of stool and urgency (95% confidence interval (CI)) among men and women aged 50–74 years (*N* = 5479)

	Total % (95 CI)	Women % (95 CI)			Men % (95 CI)		
	*N =* 5479	50-64y *n* = 1543	65-74y *n =* 1239	All *n* = 2782	50-64y *n* = 1395	65-74y *n* = 1302	All *n* = 2697
No LARS	64.1 (62.8;65.3)	58.3 (55.8;60.7)	59.1 (56.3;61.8)	58.6 (56.8;60.5)	68.2 (65.7;70.7)	71.2 (68.7;73.6)	69.7 (67.9;71.4)
Minor LARS	21.7 (20.6;22.8)	24.4 (22.3;26.7)	22.6 (20.3;25.0)	23.6 (22.0;25.2)	21.0 (18.9;23.2)	18.4 (16.3;20.6)	19.7 (18.2;21.3)
Major LARS	14.2 (13.3;15.2)	17.3 (15.4;19.3)	18.3 (16.2;20.6)	17.8 (16.4;19.2)	10.8 (9.2;12.5)	10.4 (8.8;12.2)	10.6 (9.5;11.8)
Occasions when you cannot control your flatus (wind)?^a^	25.0 (23.9;26.2)	28.7 (26.4;31.0)	31.9 (29.3;34.6)	30.1 (28.4;31.8)	16.5 (14.6;18.6)	23.2 (20.9;25.5)	19.7 (18.2;21.3)
Accidental leakage of liquid stool?^a^	3.2 (2.7;3.7)	3.2 (2.4;4.2)	4.0 (2.9;5.2)	3.5 (2.9;4.3)	3.0 (2.2;4.0)	2.6 (1.8;3.6)	2.8 (2.2;3.5)
Often or rare opening of your bowels?^b^	24.1 (23.0;25.2)	27.6 (25.4;29.9)	27.0 (24.5;29.5)	27.3 (25.7;29.0)	19.0 (17.0;21.2)	22.6 (20.4;25.0)	20.8 (19.2;22.3)
Have to open your bowels again within one hour of the last bowel opening?^a^	12.1 (11.3;13.0)	13.3 (11.6;15.1)	16.4 (14.4;18.6)	14.7 (13.4;16.0)	10.3 (8.8;12.0)	8.7 (7.3;10.4)	9.5 (8.5;10.7)
Such a strong urge to open your bowels that you have to rush to the toilet?^a^	13.7 (12.8;14.7)	16.6 (14.8;18.6)	15.7 (13.7;17.8)	16.2 (14.8;17.6)	13.0 (11.2;14.8)	9.2 (7.7;10.9)	11.2 (10.0;12.4)

^a^Proportion experiencing this symptom more than once per week

^b^Proportion opening their bowels more than 7 times a day, 4–7 times a day or less than once a day

### The patient assessment of constipation-symptoms (PAC-SYM)

Overall, the median PAC-SYM score was 0.33 (IQR: 0.17;0.75), the median abdominal score was 0.50 (IQR: 0.00;1.00), median rectal score 0.00 (IQR:0.00;0.33) and median stool score 0.40 (IQR: 0.00;0.80) (Table 4). Of the abdominal symptoms, bloating in the abdomen was the most frequent symptom (reported by 6.5% (95% CI: 5.8;7.2)). Rectal symptoms were rarely experienced (ranging from being reported by 1.2 (95% CI: 0.9;1.5) to 1.7% (95% CI: 1.4;2.1)) and of the stool symptoms, the most frequently experienced was straining to try to pass bowel movements (reported by 4.6% (95% CI: 4.0;5.2)) (Table 4).

**Table 4:** Patient Assessment of Constipation-Symptoms (PAC-SYM) score (inter quartile range (IQR)) and proportion of respondents experiencing symptoms of constipation within two weeks (95% confidence interval (CI)) among men and women aged 50–74 years (*N* = 4086)

	Total % (95% CI)	Women % (95% CI)			Men % (95% CI)		
	*N =* 4086	50-64y *n* = 1092	65-74y *n* = 969	All *n* = 2061	50–64 y *n* = 997	65-74y *n* = 1028	All *n* = 2025
**PAC-SYM score** median (IQR)	0.33 (0.17;0.75)	0.50 (0.25;0.92)	0.33 (0.17;0.75)	0.42 (0.17;0.83)	0.33 (0.08;0.67)	0.33 (0.08;0.58)	0.33 (0.08;0.67)
**Abdominal score** median (IQR)	0.50 (0.00;1.00)	0.50 (0.00;1.25)	0.25 (0.00;0.75)	0.50 (0.25;1.00)	0.50 (0.00;1.00)	0.25 (0.00;0.75)	0.25 (0.00;0.75)
**Rectal score** median (IQR)	0.00 (0.00;0.33)	0.00 (0.00;0.67)	0.00 (0.00;0.33)	0.00 (0.00;0.33)	0.00 (0.00;0.33)	0.00 (0.00;0.33)	0.00 (0.00;0.33)
**Stool score** median (IQR)	0.40 (0.00;0.80)	0.60 (0.20;1.00)	0.40 (0.00;1.00)	0.40 (0.20;1.00)	0.40 (0.00;0.80)	0.40 (0.00;0.80)	0.40 (0.00;0.80)
**Abdominal symptoms**^a^							
Discomfort in your abdomen	2.2 (1.9;2.7)	3.4 (2.6;4.5)	2.0 (1.3;3.0)	2.8 (2.2;3.5)	1.8 (1.2;2.6)	1.5 (0.9;2.4)	1.7 (1.2;2.2)
Pain in your abdomen	2.2 (1.8;2.6)	3.4 (2.5;4.4)	2.0 (1.3;3.0)	2.8 (2.2;3.5)	1.6 (1.0;2.4)	1.5 (0.9;2.4)	1.6 (1.1;2.1)
Bloating in your abdomen	6.5 (5.8;7.2)	11.0 (9.5;12.7)	5.5 (4.3;7.0)	8.6 (7.6;9.7)	5.3 (4.2;6.6)	3.3 (2.4;4.5)	4.3 (3.6;5.2)
Stomach cramps	1.7 (1.4;2.1)	2.7 (2.0;3.7)	1.4 (0.8;2.2)	2.1 (1.6;2.7)	1.6 (9.9;2.4)	1.0 (0.5;1.7)	1.3 (0.9;1.8)
**Rectal symptoms**^a^							
Painful bowel movements	1.2 (0.9;1.5)	1.7 (1.1;2.5)	1.4 (0.8;2.2)	1.5 (1.1;2.1)	0.9 (0.5;1.6)	0.6 (0.3;1.2)	0.8 (0.5;1.2)
Rectal burning during or after a bowel movement	1.7 (1.4;2.1)	2.3 (1.6;3.1)	1.5 (0.9;2.3)	1.9 (1.4;2.5)	2.0 (1.3;2.9)	1.1 (0.6;1.8)	1.6 (1.1;2.1)
Rectal bleeding or tearing during or after a bowel movement	1.4 (1.1;1.8)	2.2 (1.5;3.1)	1.2 (0.7;2.0)	1.8 (1.3;2.3)	1.5 (0.9;2.3)	0.7 (3.2;1.3)	1.1 (0.8;1.6)
**Stool symptoms**^a^							
Incomplete bowel movement, like you didn’t “finish”	3.4 (2.9;3.9)	4.9 (3.8;6.0)	3.6 (2.6;4.7)	4.3 (3.6;5.1)	3.4 (2.5;4.5)	1.5 (0.9;2.4)	2.5 (2.0;3.2)
Bowel movements that were too hard	2.9 (2.5;3.4)	4.3 (3.3;5.4)	4.0 (3.0;5.3)	4.2 (3.5;5.0)	1.5 (0.9;2.3)	1.6 (1.0;2.4)	1.6 (1.1;2.1)
Bowel movements that were too small	2.0 (1.7;2.4)	3.2 (2.4;4.2)	1.9 (1.2;2.8)	2.6 (2.1;3.3)	1.6 (1.0;2.4)	1.2 (0.6;1.9)	1.4 (1.0;1.9)
Straining or squeezing to try to pass bowel movements	4.6 (4.0;5.2)	7.3 (6.1;8.7)	4.8 (3.7;6.2)	6.2 (5.3;7.2)	2.9 (2.1;3.9)	2.9 (2.1;4.0)	2.9 (2.3;3.6)
Feeling like you have to pass a bowel movement but you couldn’t (false alarm)	2.5 (2.1;2.9)	3.2 (2.4;4.2)	3.1 (2.2;4.2)	3.2 (2.5;3.9)	1.9 (1.2;2.7)	1.6 (1.0;2.5)	1.7 (1.3;2.3)

^a^Proportion experiencing the symptoms as serious or very serious

### Associations between abdominal symptoms and personal characteristics

A greater proportion of women than men reported alarm symptoms at least once a week (PR: 1.25 (95% CI: 1.13;1.38)). The older age group (65–74 years) was less likely to experience alarm symptoms at least once a week compared to the age group 50–64 years (PR: 0.72 (95% CI: 0.65;0.81)). Furthermore, those cohabiting were less likely to experience alarm symptoms than those living alone (PR: 0.86 (95% CI: 0.76;0.96) as were those with high income compared to those with low income (PR: 0.87 (95% CI: 0.75;1.00)). Finally, both western and non-western immigrants were more likely to experience alarm symptoms than native Danes (PR: 1.21 (95% CI: 0.89;1.63) and PR: 1.31 (95% CI: 0.92;1.87), respectively) (Table 5).

**Table 5:** Prevalence ratios (PR) (95% confidence intervals ()) for alarm symptoms, Low Anterior Resection Syndrome (LARS) and Patient Assessment of Constipation-Symptoms (PAC-SYM) score

	Alarm symptoms^a^ *N =* 5488		LARS^b^ *N =* 5479	PAC_SYM^c^ *N =* 4086		
	Unadjusted PR (95% CI)	Adjusted PR^d^ (95% CI)	Unadjusted PR (95% CI)	Adjusted PR^d^ (95% CI)	Unadjusted PR (95% CI)	Adjusted PR^d^ (95% CI)
**Sex**						
Men	1 (ref)	1 (ref)	1 (ref)	1 (ref)	1 (ref)	1 (ref)
Women	1.28 (1.16;1.41)	1.25 (1.13;1.38)	1.36 (1.27;1.47)	1.34 (1.24;1.44)	1.43 (1.31;1.55)	1.39 (1.28; 1.51)
**Age (years)**						
50–64	1 (ref)	1 (ref)	1 (ref)	1 (ref)	1 (ref)	1 (ref)
65–74	0.73 (0.66;0.81)	0.72 (0.65;0.81)	0.94 (0.87;1.01)	0.93 (0.86;1.00)	0.79 (0.73;0.86)	0.77 (0.71;0.84)
**Origin**						
Danish	1 (ref)	1 (ref)	1 (ref)	1 (ref)	1 (ref)	1 (ref)
Western	1.22 (0.91;1.64)	1.21 (0.89;1.63)	0.87 (0.67;1.13)	0.90 (0.69;1.17)	1.04 (0.80;1.36)	1.01 (0.77;1.32)
Non-western	1.56 (1.12;2.16)	1.31 (0.92;1.87)	0.88 (0.62;1.25)	0.88 (0.62;1.25)	1.25 (0.91;1.70)	1.14 (0.83;1.59)
**Marital status**						
Living alone	1 (ref)	1 (ref)	1 (ref)	1 (ref)	1 (ref)	1 (ref)
Cohabiting	0.80 (0.72;0.90)	0.86 (0.76;0.96)	0.86 (0.79;0.93)	0.91 (0.83;0.99)	0.90 (0.82;0.98)	0.97 (0.88;1.07)
**Education (years)**						
≤ 10	1 (ref)	1 (ref)	1 (ref)	1 (ref)	1 (ref)	1 (ref)
11–15	0.97 (0.85;1.10)	0.97 (0.85;1.10)	0.99 (0.90;1.08)	1.01 (0.92;1.11)	0.89 (0.81;0.99)	0.90 (0.81;1.00)
> 15	0.86 (0.75;1.00)	0.86 (0.74;1.00)	0.99 (0.90;1.10)	1.00 (0.89;1.11)	0.88 (0.79;0.99)	0.88 (0.78;0.99)
**Income**^**e**^						
Low tertile	1 (ref)	1 (ref)	1 (ref)	1 (ref)	1 (ref)	1 (ref)
Middle tertile	0.91 (0.81;1.03)	0.93 (0.82;1.06)	0.90 (0.83;0.99)	0.93 (0.85;1.02)	0.91 (0.83;1.01)	0.92 (0.83;1.03)
High tertile	0.85 (0.75;0.96)	0.87 (0.75;1.00)	0.89 (0.81;0.97)	0.92 (0.83;1.02)	0.86 (0.78;0.95)	0.86 (0.77;0.97)

^a^Not experiencing any alarm symptoms at least once a week vs. experiencing at least one alarm symptom at least once a week

^b^Experiencing No LARS vs. experiencing minor or major LARS

^c^The two thirds with lowest score vs. the one third with highest score

^d^Adjusted for the remaining demographic and socioeconomic factors.

^e^OECD-adjusted household income: see methods.

Women were more likely than men to report minor or major LARS (PR: 1.34 (95% CI: 1.24;1.44). Even though not statistically significant, western and non-western immigrants were less likely to report minor or major LARS than native Danes (PR: 0.90 (95% CI: 0.69;1.17) and PR: 0.88 (95% CI: 0.62;1.25), respectively. Finally, those cohabiting were less likely than those living alone to report minor or major LARS (PR: 0.91 (95% CI: 0.83;0.99)) (Table 5).

Women were more likely to have high mean overall PAC-SYM score than men (PR: 1.39 (95% CI: 1.28;1.51)). The older age group was less likely to have high mean score than the younger (PR: 0.77 (95% CI: 0.71;0.84)) as were those with high education compared to low and high income compared to low (PR: 0.88 (95% CI: 0.78;0.99) and PR: 0.86 (95% CI: 0.77;0.97), respectively) (Table 5).

## Discussion

### Main findings

Overall, abdominal symptoms were commonly experienced among CRC screening eligible men and women. Abdominal pain at least once a week and unexplained tiredness within the last 4 weeks were the most commonly experienced alarm symptoms reported by 13.1 and 12.0%, respectively. Also, 14.2% experienced major LARS. As many as one third of those experiencing alarm symptoms for more than 1 month had not consulted a doctor. In general, symptoms were more often experienced by women than men and more often in the younger age group.

### Strengths and limitations

One of the main strengths of the study was the large study population enabling sub-group analyzes. Further, the study population consisted of all invited residents over a normal three-week period in the screening program thus representing a general sample of screening eligible men and women. However, only 49% responded to the questionnaire inducing a risk of selection bias. Non-responders were more likely men, of foreign origin, had lower education and income level. It is likely that the proportion of abdominal symptoms in these groups are higher than among respondents, potentially causing underestimation of the true prevalence in the general population. This is especially worth considering in the group of elderly men with the lowest response rate. Further, survey data may be influenced by recall and social desirability bias. Abdominal symptoms may be associated with some taboo causing individuals with symptoms not to answer or to underestimate the symptoms. On the other hand, individuals with symptoms could be more likely to answer an anonymous survey.

The use of web-based questionnaires may influence the response rate in older age groups such as those included in this study. However, the mandatory digital communication with any Danish public administration may minimize this selection bias as the respondents are used to navigate in digital communication.

Due to the design of the study in a representative region of Denmark, we assume that the results can be reproduced in all of Denmark. The results may also be generalizable to countries with similar demographics, health care systems and equivalent health conditions among the elderly citizens.

### Interpretation of results

The occurrence of minor and major LARS measured in our study was similar to findings in the existing literature using the original answering categories [24]. In our study, the general prevalence of major LARS was 14.1% compared to 14.0% in the mentioned study with at study population aged 50–79 years. Compared to the existing literature, our study found a slightly lower prevalence for major LARS in women. For men, we found a slightly higher prevalence of major LARS compared to the existing literature.

For PAC-SYM, no previous literature on normative data in general populations were found. Instead, recent studies describe data of patient groups. In our study, the mean PAC-SYM was 0.52. In a review from 2017 focusing on patients with functional constipation, the reported mean was 1.70, which is of course higher given the focus on patients with constipation [25]. The results from our study represent the first published normative data on PAC-SYM. Hence, it could be used as a reference population in future studies.

The findings that symptom experiences were more frequent among women than men and, especially for women, more frequent among younger than older age groups are in line with another Danish study by Rasmussen et al. reporting symptoms for men and women aged 40–79 years [5]. However, in their study the proportion reporting symptoms is higher than in our study. In our sensitivity analysis, the proportions were more in line with those reported by Rasmussen et al. This indicates that the overall differences are primarily explained by the fact that Rasmussen et al. asked if respondents had experienced any of the given abdominal symptoms within the past 4 weeks whereas we asked if the symptoms were experienced more than once a week. Women are known to live longer than men but report poorer health status [26, 27]. This is consistent with our finding that women experienced more symptoms than men. This may be explained by a male tendency of under-reporting health problems but also by differences in social roles and health behavior. It may be culturally more acceptable for women to be sick, report more health problems and get advice about illness, suggesting that gender differences in health could be partially attributed to gender role expectations [26, 27]. Finally, there are differences in health-care utilization and help-seeking behavior among men and women. This is consistent with our finding that more men than women report having had symptoms for 1 month or longer. Likewise, a greater proportion of those reporting that they have not seen a general practitioner regarding their abdominal symptoms seemed to be men [28, 29].

The fact that symptoms were more frequently experienced in the younger age group is described as the paradox of aging where older people report greater mental health and well-being than younger people. This may be associated with a gradual change in attitude including higher acceptance of one’s physical limitations and a more realistic appraisal of one’s own strengths and limitations [30]. Thus, this may be more a sign of differences in expectations to symptoms and not the actual presence of symptoms.

Taking our results into consideration, it is important to emphasize when communicating with the citizens that alarm symptoms should result in contacting a physician whereas it may be relevant to participate in screening with other kinds of minor discomfort from the stomach. Even though alarm symptoms of CRC have low positive predictive values of cancer, it is concerning that so many respondents experiencing alarm symptoms more than once a week for longer than 1 month have not consulted a physician. Even though rarely experienced, it is critical that less than half of those experiencing very dark/black stool and two thirds of those experiencing fresh blood in the stool has consulted a physician since rectal bleeding is one of the symptoms with highest positive predictive value for CRC cancer [7, 8]. Future research is needed to address whether citizens experiencing symptoms will be more likely to test positive in CRC screening.

## Conclusion

The cross-sectional findings in this study illustrate that abdominal symptoms were frequent among screening-eligible men and women aged 50–74 years and possibly influenced by many individual and social factors. This should be taken into account when implementing and improving CRC screening strategies. A concerning high number of respondents experiencing alarm symptoms for more than 1 month had not consulted a doctor. This calls for attention to abdominal symptoms in general and how those with abdominal symptoms should participate in CRC screening.

## Supplementary Information

**Additional file 1: Appendix 1.** Questions and response categories in the survey data on abdominal symptoms.

## Data Availability

The data that support the findings of this study are available from Statistics Denmark and the Danish Health Data Authority but restrictions apply to the availability of these data, which were used under license for the current study. The participants did not consent to publication of the survey data generated in the current study but data may be available in anonymous form from the corresponding author upon reasonable request.

## Abbreviations

CI: Confidence interval
CRC: Colorectal cancer
IQR: Inter quartile range
LARS: Lower Anterior Resection Syndrome
OECD: Organisation for Economic Co-operation and Development
PAC-SYM: Patient Assessment of Constipation-Symptoms
PR: Prevalence rate
REDCap: Research Electronic Data Capture
UNESCO: United Nations Educational, Scientific and Cultural Organization
