# Supplementary material for: Prevalence of self-reported abdominal symptoms among 50–74-years-old men and women eligible for colorectal cancer screening –a cross-sectional study
Source: BMC Cancer. 2021 Aug 10;21:910. doi: 10.1186/s12885-021-08657-z (PMC8356437; doi:10.1186/s12885-021-08657-z)
Supplement: Supplementary file 1 — Additional file 1: Appendix 1. Questions and response categories in the survey data on abdominal symptoms. [file 12885_2021_8657_MOESM1_ESM.docx]

**Appendix I** Questions and response categories in the survey data on abdominal symptoms

| **Question** | | **Answering categories** |
| --- | --- | --- |
| Ad hoc questions | | |
| Have you experienced abdominal pain?  Have you experienced mucus in your stool?  Have you experienced fresh blood in your stool?  Have you experienced very dark/black stool? | | Never  Less than once a month  Once a month  More than once a month but not every week  At least once a week but not every day*  At least once a day* |
|  | |  |
| Have you been unusually tired for no reason within the last four weeks?  Have you experienced unexplained weight loss during the last months? | | Not at all  A little  Some  A lot |
| Low Anterior Resection Syndrome Score (LARS) | | |
| Do you ever have occasions when you cannot control your flatus (wind)?  Do you ever have any accidental leakage of liquid stool? | | Never  Less than once a month  Once a month  More than once a month but not every week  At least once a week but not every day  At least once a day |
|  | |  |
| How often do you open your bowels? | | More than 7 times a day  4-7 times a day  1-3 times a day  Less than once a day |
|  | |  |
| Do you ever have to open your bowels again within one hour of the last bowel opening?  Do you ever have such a strong urge to open your bowels that you have to rush to the toilet? | | Never  Less than once per week  More than once per week |
| The Patient Assessment of Constipation-Symptoms (PAC-SYM) | | |
| *Stool symptoms:* |  | Absent  Mild  Moderate  Severe  Very severe |
| Discomfort in your abdomen  Pain in your abdomen  Bloating in your abdomen  Stomach cramps | |  |
| *Rectal symptoms:* | |  |
| Painful bowel movements  Rectal burning during or after a bowel movement  Rectal bleeding or tearing during or after a bowel movement | |  |
| *Abdominal symptoms:* | |  |
| Incomplete bowel movement, like you didn’t “finish”  Bowel movements that were too hard  Bowel movements that were too small  Straining or squeezing to try to pass bowel movements  Feeling like you have to pass a bowel movement  but you couldn’t (false alarm) | |  |

*****If using this category, respondents were asked to state how long the symptom has been present: Less than one month, 1-6 months, more than 6 months, do not remember.
